# Vague Congruences and Quotient Lattice Implication Algebras

**DOI:** 10.1155/2014/197403

**Published:** 2014-07-14

**Authors:** Xiaoyan Qin, Yi Liu, Yang Xu

**Affiliations:** ^1^Intelligent Control Development Center, Southwest Jiaotong University, Chengdu, Sichuan 610031, China; ^2^College of Mathematics and Computer Science, Shanxi Normal University, Linfen, Shanxi 041004, China; ^3^Key Laboratory of Numerical Simulation, Sichuan Provincial College, Neijiang Normal University, Neijiang, Sichuan 641000, China

## Abstract

The aim of this paper is to further develop the congruence theory on lattice implication algebras. Firstly, we introduce the notions of vague similarity relations based on vague relations and vague congruence relations. Secondly, the equivalent characterizations of vague congruence relations are investigated. Thirdly, the relation between the set of vague filters and the set of vague congruences is studied. Finally, we construct a new lattice implication algebra induced by a vague congruence, and the homomorphism theorem is given.

## 1. Introduction

Artificial intelligence (AI) is a newly developed and highly comprehensive frontier science with rapid development, and it mainly studies how to simulate human intelligence behavior by machine. Intelligent information processing is an important direction in the field of AI. Classical mathematical logic is enough to deal with reasoning in traditional mathematical theory, while, in the real world, there are large numbers of problems with uncertainties. To simulate human intelligence behavior better, uncertainty reasoning becomes a key part of computational science and artificial intelligence, and its rationality should be based on foundation of a kind of scientific logic, which is called nonclassical logic [1–3]. Therefore, nonclassical logic has become a considerable formal tool for computer science and artificial intelligence to deal with fuzzy information and uncertain information. Many-valued logic is an extension and development of classical logic and has always been a crucial direction in nonclassical logic.

As an important many-valued logic, lattice-valued logic [4] has two prominent roles. One is to extend the chain-type truth-valued field of the current logics to some relatively general lattices. The other is that the incompletely comparable property of truth value characterized by the general lattice can more effectively reflect the uncertainty of human being's thinking, judging, and decision. Hence, lattice-valued logic has become an important research field and strongly influenced the development of algebraic logic, computer science, and artificial intelligent technology. In order to provide a reliable logical foundation for uncertain information processing theory, especially for the fuzziness and the incomparability in uncertain information reasoning, Xu [5] combined algebraic lattice and implication algebra, proposed the concept of lattice implication algebras (LIAs for short), and discussed some properties. Since then, this logical algebra has been extensively investigated by several researchers [4–14].

In 1965, Zadeh introduced the concept of fuzzy set [15]. So far, this idea has been applied to other algebraic structures such as groups, semigroups, rings, modules, vector spaces, and topologies and widely used in many fields. Meanwhile, the deficiency of fuzzy sets is also attracting the researchers' attention. For example, a fuzzy set is a single function, and it cannot express the evidence of supporting and opposing. For this reason, the concept of vague set [16] was introduced in 1993 by Gau and Buehrer. In a vague set *A*, there are two membership functions: a truth membership function *t*_*A*_ and a false membership function *f*_*A*_, where *t*_*A*_(*x*) is a lower bound of the grade of membership of *x* derived from the “evidence for *x*” and *f*_*A*_(*x*) is a lower bound on the negation of *x* derived from the “evidence against *x*” and *t*_*A*_(*x*) + *f*_*A*_(*x*) ≤ 1. Thus, the grade of membership in a vague set *A* is a subinterval [*t*_*A*_(*x*), 1 − *f*_*A*_(*x*)] of [0,1]. The idea of vague sets is an extension of fuzzy sets so that the membership of every element can be divided into two aspects including supporting and opposing. In fact, the idea of vague set is the same with the idea of intuitionistic fuzzy set [17]; so, the vague set is equivalent to intuitionistic fuzzy set.

With the development of vague set theory, some structures of algebras corresponding to vague set have been studied. Biswas [18] initiated the study of vague algebras by studying vague groups. Eswarlal [19] studied the vague ideals and normal vague ideals in semirings. Kham et al. [20] studied the vague relation and its properties, and moreover intuitionistic fuzzy filters and intuitionistic fuzzy congruences in a residuated lattice were researched [10, 13, 21–24]. However, it is well known that lattice implication algebra is a special kind of residuated lattice; so, lattice implication algebras have some special properties which are not possessed by a general residuated lattice. Therefore, it is necessary to investigate the vague congruence relation in a lattice implication algebra. Moreover, quotient algebras are a basic tool for exploring the structures of algebras. There are close correlations among filters, congruences, and quotient algebras.

In this paper, we introduce the concept of the vague congruence relation in the lattice implication algebra. In Section 2, we recall some definitions and theorems which will be used in later sections of this paper. In Section 3, we propose the concepts of vague similarity relation based on vague relation and vague congruence relations, and furthermore some properties and equivalent characterizations of vague congruence relation are investigated. Moreover, we show that there is a one-to-one correspondence between the set of all vague filters and all vague congruences of a lattice implication algebra. In Section 4, we construct a new lattice implication algebra induced by vague congruences, and finally the homomorphism theorem is given.

## 2. Preliminaries

**Definition 1 (see [5]).**

Let (*L*, ∨, ∧, *O*, *I*) be a bounded lattice with an order-reversing involution ′, the greatest element *I* and the smallest element *O*, and let →:*L* × *L* → *L* be a mapping. *L* = (*L*, ∨, ∧, ′, →, *O*, *I*) is called a lattice implication algebra if the following conditions hold for any *x*, *y*, *z* ∈ *L*.

(*I*_1_ ) *x* → (*y* → *z*) = *y* → (*x* → *z*).
( *I*_2_ ) *x* → *x* = *I*.
( *I*_3_ ) *x* → *y* = *y*′ → *x*′.
( *I*_4_ ) *x* → *y* = *y* → *x* = *I* implies *x* = *y*.
( *I*_5_ ) (*x* → *y*) → *y* = (*y* → *x*) → *x*.
( *l*_1_ ) (*x*∨*y*) → *z* = (*x* → *z*)∧(*y* → *z*).
( *l*_2_ ) (*x*∧*y*) → *z* = (*x* → *z*)∨(*y* → *z*).

In this paper, denote *L* as a lattice implication algebra (*L*, ∨, ∧, ′, →, *O*, *I*).

In the following, we will list some basic properties of lattice implication algebras, and they are useful for our subsequent work.

**Theorem 2 (see [4]).**

Let *L* be a lattice implication algebra. Then, for any *x*, *y*, *z* ∈ *L*, the following conclusions hold.

1. If *I* → *x* = *I*, then *x* = *I*.
2. *I* → *x* = *x* and *x* → *O* = *x*′.
3. *O* → *x* = *I* and *x* → *I* = *I*.
4. (*x* → *y*)→((*y* → *z*)→(*x* → *z*)) = *I*.
5. (*x* → *y*)∨(*y* → *x*) = *I*.
6. If *x* ≤ *y*, then *x* → *z* ≥ *y* → *z* and *z* → *x* ≤ *z* → *y*.
7. *x* ≤ *y* if and only if *x* → *y* = *I*.
8. (*z* → *x*)→(*z* → *y*) = (*x*∧*z*) → *y* = (*x* → *z*)→(*x* → *y*).
9. *x* → (*y* → *z*) = (*x*∨*y*) → *z* if and only if *x* → (*y* → *z*) = *x* → *z* = *y* → *z*.
10. *z* ≤ *y* → *x* if and only if *y* ≤ *z* → *x*.

**Definition 3 (see [12]).**

A nonempty subset *F* of a lattice implication algebra *L* is called a* filter* of *L* if it satisfies the following.

(F1) *I* ∈ *F*.
(F2) (∀*x* ∈ *F*) (∀*y* ∈ *L*) (*x* → *y* ∈ *F*⇒*y* ∈ *F*).

**Definition 4 (see [16]).**

A vague set *A* in the universe of discourse *X* is characterized by two membership functions given by

1. a truth membership function *t*_*A*_ : *X* → [0,1],
2. a false membership function *f*_*A*_ : *X* → [0,1],

where *t*_*A*_(*x*) is a lower bound of the grade of membership of *x* derived from the “evidence for *x*” and *f*_*A*_(*x*) is a lower bound on the negation of *x* derived from the “evidence against *x*” and *t*_*A*_(*x*) + *f*_*A*_(*x*) ≤ 1. Thus, the grade of membership of *x* in the vague set *A* is bounded by a subinterval [*t*_*A*_(*x*), 1 − *f*_*A*_(*x*)] of [0,1]. The vague set *A* is written as follows:

(1) $$\begin{array}{c} A=\left\{\langle x,\left[t_{A}\left(x\right),f_{A}\left(x\right)\right]\rangle \;|\;x\in X\right\}, \end{array}$$

where the interval [*t*_*A*_(*x*), 1 − *f*_*A*_(*x*)] is called the value of *x* in the vague set *A* and denoted by *V*_*A*_(*x*).

**Definition 5 (see [16]).**

A Vague set *A* is contained in the other vague set *B*, *A*⊆*B* if and only if *V*_*A*_(*x*) ≤ *V*_*B*_(*x*); that is, *t*_*A*_(*x*) ≤ *t*_*B*_(*x*) and 1 − *f*_*A*_(*x*) ≤ 1 − *f*_*B*_(*x*) for any *x* ∈ *X*.

**Definition 6 (see [16]).**

Let *A* be a vague set of *X* with the truth membership function *t*_*A*_ and the false membership function *f*_*A*_. For any *α*, *β* ∈ [0,1], the (*α*, *β*)-cut of the vague set *A* is a crisp subset *A*_(*α*,*β*)_ of the set *X* by

(2) $$\begin{array}{c} A_{\left(\alpha ,\beta \right)}=\left\{x\in X\;|\;V_{A}\left(x\right)\geq \left[\alpha ,\beta \right]\right\}. \end{array}$$

Obviously, *A*_(0,0)_ = *X*.

**Definition 7 (see [16]).**

Let *A* be a vague set of *X* with the truth membership function *t*_*A*_ and the false membership function *f*_*A*_. For any *α* ∈ [0,1], the (*α*, *α*)-cut of the vague set *A* is a crisp subset *A*_*α*_ = *A*_(*α*,*α*)_.

Let *A* be a vague set of *X*, *A*_0_ = *X*; and if *α* ≤ *β*, then *A*_*α*_⊆*A*_*β*_ and *A*_(*α*,*β*)_ = *A*_*α*_. Equivalently, we can define the *α*-cut *A*_*α*_ of *A*, *A*_*α*_ = {*x* ∈ *X*∣*t*_*A*_(*x*) ≥ *α*}.

Let *A* and *B* be two vague sets of *X*; then, *A* = *B* if and only if *V*_*A*_(*x*) = *V*_*B*_(*x*) for any *x* ∈ *X*.

Let *I*[0,1] denote the family of all closed subintervals of [0,1]. If *I*_1_ = [*a*_1_, *b*_1_] and *I*_2_ = [*a*_2_, *b*_2_] are two elements of *I*[0,1], we call *I*_1_ ≥ *I*_2_ if *a*_1_ ≥ *a*_2_ and *b*_1_ ≥ *b*_2_. We define the term imax to mean the maximum of two intervals as follows:

(3) $$\begin{array}{c} \text{imax}\left\{I_{1},I_{2}\right\}=\left[\max\left\{a_{1},a_{2}\right\},\max\left\{b_{1},b_{2}\right\}\right]. \end{array}$$

Similarly, we can define the term imin of any two intervals.

**Definition 8 (see [20]).**

Let *X* and *Y* be two universes. A vague relation of the universe *X* with the universe *Y* is a vague set of the Cartesian product *X* × *Y*.

**Definition 9 (see [20]).**

Let *X* and *Y* be two universes. A vague subset *R* of discourse *X* × *Y* is characterized by two membership functions given by the following:

1. a truth membership function *t*_*R*_ : *X* × *Y* → [0,1],
2. a false membership function *f*_*R*_ : *X* × *Y* → [0,1],

where *t*_*R*_(*x*, *y*) is a lower bound of the grade of membership of (*x*, *y*) derived from the “evidence for (*x*, *y*)” and *f*_*R*_(*x*, *y*) is a lower bound on the negation of (*x*, *y*) derived from the “evidence against (*x*, *y*)” and *t*_*R*_(*x*, *y*) + *f*_*R*_(*x*, *y*) ≤ 1. Thus, the grade of membership of (*x*, *y*) in the vague set *R* is bounded by a subinterval [*t*_*R*_(*x*, *y*), 1 − *f*_*R*_(*x*, *y*)] of [0,1]. The vague relation *R* is written as follows:

(4) $$\begin{array}{c} R=\left\{\langle \left(x,y\right),\left[t_{R}\left(x,y\right),f_{R}\left(x,y\right)\right]\rangle \;|\;\left(x,y\right)\in X\times Y\right\}, \end{array}$$

where the interval [*t*_*R*_(*x*, *y*), 1 − *f*_*R*_(*x*, *y*)] is called the value of (*x*, *y*) in the vague relation *R* and denoted by *V*_*R*_(*x*, *y*).

## 3. Equivalent Characterizations of Vague Congruence Relation

From Definitions 4 and 8, we can obtain the equivalent definition of vague relation.

**Definition 10 .**

Let *X* be nonempty universe; the vague relation *R* on *X* is called vague similarity relation if *R* satisfies the following conditions:

1. ∀*x* ∈ *X*, *V*_*R*_(*x*, *x*) = sup⁡_*u*,*v*∈*X*_ *V*_*R*_(*u*, *v*) (vague reflexivity);
2. ∀*x*, *y* ∈ *X*, *V*_*R*_(*x*, *y*) = *V*_*R*_(*y*, *x*) (vague symmetric);
3. ∀*x*, *y*, *z* ∈ *X*, imin{*V*_*R*_(*x*, *z*), *V*_*R*_(*z*, *y*)} ≤ *V*_*R*_(*x*, *y*) (vague transitivity).

**Remark 11 .**

For the vague transitivity,

(5) (∀x,y,z∈X)imin{VR(x,z),VR(z,y)}≤VR(x,y)⟺(∀x,y∈X)sup⁡z∈Ximin{VR(x,z),VR(z,y)}≤VR(x,y).

**Definition 12 .**

Let *R* be a vague relation on *X*, and *λ* = [*λ*_1_, *λ*_2_] ∈ *I*[0,1], where *λ*_1_, *λ*_2_ ∈ [0,1] and *λ*_1_ ≤ *λ*_2_. If *V*_*R*_(*x*, *y*) ≥ *λ* holds for any *x*, *y* ∈ *X*, then we call the set *R*_*λ*_ = {(*x*, *y*) ∈ *X* × *X*∣*V*_*R*_(*x*, *y*) ≥ *λ*} is *λ*-level relation of *R*.

From Definition 12, any level relation *R*_*λ*_ of vague relation *R* on *X* is a relation on *X*, whose characteristic function *χ*_*R*_*λ*__ is as follows:

(6) $$\begin{array}{c} \chi_{R_{\lambda }}\left(x,y\right)=\{\begin{array}{cc} 1, & \text{if}\left(x,y\right)\in R_{\lambda },\\ 0, & \text{if}\left(x,y\right)\notin R_{\lambda }. \end{array} \end{array}$$

**Theorem 13 .**

Let *R* be a vague relation on universe *X*; then, *R* is vague similarity relation if and only if *R*_*λ*_ is an equivalent relation on *X*, where *λ* is a close subinterval of [0,1].

**Definition 14 .**

Let *R* be a vague relation on *L*. *R* is said to be a vague congruence relation on *L*, if

1. *R* is a vague similarity relation on *L*;
2. *V*_*R*_(*x* → *z*, *y* → *z*) ≥ *V*_*R*_(*x*, *y*) for any *x*, *y*, *z* ∈ *L*.

Let *R* be a vague relation on *L*, for all close subintervals *t* = [*t*_1_, *t*_2_] ∈ *I*[0,1]; the *t*-level relation and strong *t*-level subset of *L*, respectively, are defined as follows:

(7) $$\begin{array}{c} R_{t}=\left\{\left(x,y\right)\in L\times L\;|\;V_{R}\left(x,y\right)\geq t\right\},\\ R_{t^{>}}=\left\{\left(x,y\right)\in L\times L\;|\;V_{R}\left(x,y\right)>t\right\}. \end{array}$$

**Definition 15 .**

Let *R* be a vague relation on nonempty set *L*. *R* satisfies the sup⁡ property if, for every subset *T* of *L*, there exists *u*, *v* ∈ *T* such that sup⁡_*x*,*y*∈*T*_ *V*_*R*_(*x*, *y*) = *V*_*R*_(*u*, *v*).

**Lemma 16 .**

Let *R* be a vague relation on *L*; then, *R*_*t*_ = ⋂_[0,0]≤*s*<*t*_ *R*_*t*^>^_ and *R*_*t*^>^_ = ⋃_*t*<*s*≤[1,1]_ *R*_*s*_, where *t*, *s* ∈ *I*[0,1].

**Proof**

Let *R* be a vague relation on *L*, *t* ∈ *I*[0,1]; we have *R*_*t*_ = {(*x*, *y*) ∈ *L* × *L*∣*V*_*R*_(*x*, *y*) ≥ *t*} = ⋂_[0,0]≤*s*<*t*_{(*x*, *y*) ∈ *L* × *L*∣*V*_*R*_(*x*, *y*) > *s*} = ⋂_[0,0]≤*s*<*t*_ *R*_*t*^>^_. *R*_*t*^>^_ = {(*x*, *y*) ∈ *L* × *L*∣*V*_*R*_(*x*, *y*) > *t*} = ⋃_*t*<*s*≤[1,1]_{(*x*, *y*) ∈ *L* × *L*∣*V*_*R*_(*x*, *y*) ≥ *s*} = ⋃_*t*<*s*≤[1,1]_ *R*_*t*_.

**Theorem 17 .**

Let *R* be a vague relation on *L* that satisfies the sup⁡ property. Then, the following statements are equivalent:

1. *R* is a vague similarity relation on *L*;
2. *R*_*t*_(≠*∅*) is an equivalence relation on *L*, for all *t* ∈ *I*[0,1];
3. *R*_*t*^>^_(≠*∅*) is an equivalence relation on *L*, for all [0,0] ≤ *t* < [1,1].

**Proof**

((1)⇒(2)) It can be easily proved.

((2)⇒(3)) Let *R*_*t*^>^_ ≠ *∅*, for all [0,0] ≤ *t* < [1,1]. It follows by Lemma 16 that there exist *s* ∈ *I*[0,1] and *t* < *s* ≤ [1,1] such that *R*_*s*_ ≠ *∅*. Since *R*_*s*_ is an equivalent relation, *R*_*s*_ is reflexive; that is, (*x*, *x*) ∈ *R*_*s*_⊆*R*_*t*^>^_. It follows that *R*_*t*^>^_ is reflexive. Similarly, we can prove *R*_*t*^>^_ is symmetric.

Now, we need to prove *R*_*t*^>^_ is transitive. Suppose (*x*, *y*) ∈ *R*_*t*^>^_, (*y*, *z*) ∈ *R*_*t*^>^_; then, there exist *s*_1_, *s*_2_ ∈ *I*[0,1] and *t* < *s*_1_ ≤ [1,1], *t* < *s*_2_ ≤ [1,1] such that (*x*, *y*) ∈ *R*_*s*_1__, (*y*, *z*) ∈ *R*_*s*_2__. Taking *α* = imin{*s*_1_, *s*_2_}, then *α* ∈ *I*[0,1] and *t* < *α* ≤ [1,1]. Therefore, (*x*, *y*) ∈ *R*_*s*_1__⊆*R*_*α*_, (*y*, *z*) ∈ *R*_*s*_2__⊆*R*_*α*_. By (2), it follows that *R*_*α*_ is an equivalent relation, then (*x*, *z*) ∈ *R*_*α*_⊆*R*_*t*^>^_. Hence, *R*_*t*^>^_ is transitive. And so *R*_*t*^>^_ is an equivalence relation on *L*, for all [0,0] ≤ *t* < [1,1].

((3)⇒(1)) Let sup⁡_*u*,*v*∈*L*_(*V*_*R*_(*u*, *v*)) = *t* ∈ *I*[0,1]. Since *R* satisfies sup⁡ property, then there exists *a*, *b* ∈ *L* such that *V*_*R*_(*a*, *b*) = *t*, which implies that *R*_*t*_ ≠ *∅*; of course, *R*_*s*^>^_ ≠ *∅* for any *s* ∈ *I*[0,1] and [0,0] ≤ *s* < *t*. Therefore, (*x*, *x*) ∈ *R*_*s*^>^_, and so (*x*, *x*) ∈ ⋂_[0,0]≤*s*<*t*_ = *R*_*t*_. We have *V*_*R*_(*x*, *x*) ≥ *t* ≥ *V*_*R*_(*x*, *x*). Therefore, *V*_*R*_(*x*, *x*) = sup_*u*,*v*∈*L*_(*V*_*R*_(*u*, *v*)) = *t* which shows *R*_*t*^>^_ is reflexive. Similarly, we can show that *R*_*t*^>^_ is symmetric. Now, we show *R*_*t*^>^_ is transitive. Let *t* = imin{*V*_*R*_(*x*, *z*), *V*_*R*_(*z*, *y*)}, for any *x*, *y* ∈ *L* and some *z* ∈ *L*; this implies that *V*_*R*_(*x*, *z*) ≥ *t*, *V*_*R*_(*z*, *y*) ≥ *t*. Therefore, (*x*, *z*), (*z*, *y*) ∈ *R*_*t*_. By Lemma 16, we have (*x*, *z*), (*z*, *y*) ∈ *R*_*s*^>^_ for any [0,0] ≤ *s* < *t*; it follows from (3) that (*x*, *y*) ∈ *R*_*s*^>^_ for any [0,0] ≤ *s* < *t*. Hence, *V*_*R*_(*x*, *y*) ≥ *t* = imin{*V*_*R*_(*x*, *z*), *V*_*R*_(*z*, *y*)}; so, (1) holds.

**Theorem 18 .**

Let *R* be a vague relation on *L* that satisfies the sup⁡ property. Then, the following statements are equivalent:

1. *R* is a vague congruence relation on *L*;
2. *R*_*t*_(≠*∅*) is a congruence relation on *L*, for all *t* ∈ *I*[0,1];
3. *R*_*t*^>^_(≠*∅*) is a congruence relation on *L*, for all [0,0] ≤ *t* < [1,1].

**Proof**

(1)⇒(2) Let *R*_*t*_ ≠ *ϕ*, for all *t* ∈ *I*[0,1]. Since *R* is a vague congruence relation *L*, then *R*_*t*_ ≠ *ϕ* is an equivalence relation. Let (*x*, *y*) ∈ *R*_*t*_; then, *V*_*R*_(*x*, *y*) ≥ *t*. For any *z* ∈ *L*, since *R* is a vague congruence relation on *L*, then *V*_*R*_(*x* → *z*,  *y* → *z*) ≥ *V*_*R*_(*x*, *y*) ≥ *t*. That is, (*x* → *z*,  *y* → *z*) ∈ *R*_*t*_. Hence, *R*_*t*_ ≠ *ϕ* is a congruence, for all *t* ∈ *I*[0,1].

(2)⇒(3) Let *R*_*t*^>^_ ≠ *ϕ*, for *t* ∈ *I*[0,1). By Theorem 13, we know that *R*_*t*^>^_ is an equivalence relation on *L*. Let (*x*, *y*) ∈ *R*_*t*_; then there exist *s* ∈ *I*[0,1] and *t* < *s* ≤ [1,1] such that (*x*, *y*) ∈ *R*_*s*_ by Lemma 16. Since *R*_*s*_ is a congruence relation on *L*, then (*x* → *z*,  *y* → *z*) ∈ *R*_*s*_⊆*R*_*t*^>^_. That is, (*x* → *z*,  *y* → *z*) ∈ *R*_*t*^>^_. Hence, *R*_*t*^>^_ is a congruence on *L* for *t* ∈ *I*[0,1).

(3)⇒(1) We know that *R* is a vague similarity relation on *L* from Lemma 16.

Suppose that *x*, *y*, *z* ∈ *L* and *V*_*R*_(*x*, *y*) = *t*, then (*x*, *y*) ∈ *R*_*t*_ = ⋂_[0,0]≤*s*<*t*_ *ρ*_*s*^>^_ by Lemma 16. Since *R*_*s*^>^_ is a congruence relation on *L*, then (*x* → *z*,  *y* → *z*) ∈ *R*_*s*^>^_. Thus, (*x* → *z*,  *y* → *z*) ∈ ⋂_[0,0]≤*s*<*t*_ *R*_*s*^>^_ = *R*_*t*_. That is, *V*_*R*_*t*__(*x* → *z*,  *y* → *z*) ≥ *t* = *V*_*R*_(*x*, *y*). Hence, *R* is a vague congruence relation on *L*.

## 4. Quotient LIAs and Homomorphism Theorem

Let *R* be a vague similarity relation on *L*. For each *a* ∈ *L*, we define a vague subset *R* ^*a*^ = {(*x*, [*t*_*R*^*a*^_(*x*), *f*_*R*^*a*^_(*x*)]∣*x* ∈ *L*} of *L*, where *V*_*R*^*a*^_(*x*) = *V*_*R*_(*a*, *x*). for all *x* ∈ *L*.

**Proposition 19 .**

Let *R* be a vague congruence on *L*; then, *R* ^*I*^ is a vague filter of *L*.

**Proposition 20 .**

Let *R* be a vague congruence on *L*. For any *a*, *b* ∈ *L*, we have

(8) $$\begin{array}{c} R^{a}=R^{b}\;iff\;\;V_{R}\left(a,b\right)=\underset{y,z\in L}{\sup}V_{R}\left(y,z\right), \end{array}$$

**Proof**

Let *R* ^*a*^ = *R* ^*b*^; then, *V*_*R*^*a*^_ = *V*_*R*^*b*^_. Since *R* is a vague congruence on *L*, then

(9) $$\begin{array}{c} V_{R}\left(a,b\right)=V_{R^{a}}\left(b\right)=V_{R^{b}}\left(b\right)=V_{R}\left(b,b\right)=\underset{y,z\in L}{\sup}\rho \left(y,z\right). \end{array}$$

Conversely, assume *V*_*R*_(*a*, *b*) = sup⁡_*y*,*z*∈*L*_ *ρ*(*y*, *z*). For any *x* ∈ *L*, since *V*_*R*^*a*^_(*x*) = *V*_*R*_(*a*, *x*) ≥ sup⁡_*m*∈*L*_imin{*V*_*R*_(*a*, *m*), *V*_*R*_(*m*, *x*)} ≥ imin{*V*_*R*_(*a*, *b*), *V*_*R*_(*b*, *x*)} = *V*_*R*_(*b*, *x*) = *V*_*R*^*b*^_(*x*), then *V*_*R*^*a*^_⊇*V*_*R*^*b*^_. Similarly, we have *V*_*R*^*a*^_⊆*V*_*R*^*b*^_. Hence, *V*_*R*^*a*^_ = *V*_*R*^*b*^_, and so *R* ^*a*^ = *R* ^*b*^.

**Definition 21 .**

Let *R* be a vague congruence on a lattice implication algebra *L* and let *a* ∈ *L*. The vague subset *R* ^*a*^ of *L* is called a vague congruence class of *R* containing *a* ∈ *L*.

Denote

(10) $$\begin{array}{c} \frac{L}{R}=\left\{R^{a}a\in L\right\}, \end{array}$$

which is the set of vague congruence classes of *L* w.r.t. *R*.

In *L*/*R*, we define the operations as follows:

(11) $$\begin{array}{c} R^{a}⟶R^{b}=R^{a\to b};\\ R^{a}\cup R^{b}=R^{a\vee b};\\ R^{a}\cap R^{b}=R^{a\wedge b};\\ {\left(R^{a}\right)}^{\prime }=R^{a^{\prime }}. \end{array}$$

**Proposition 22 .**

Let *L* be a lattice implication algebra. The operations →, ∪, ∩, ′ on *L*/*R* are well defined.

**Proof**

Suppose that *R* ^*a*^ = *R* ^*b*^ and *R* ^*c*^ = *R* ^*d*^. Then, we have that *V*_*R*_(*a*, *b*) = *V*_*R*_(*c*, *d*) = sup⁡_*y*,*z*∈*L*_ *V*_*R*_(*y*, *z*) by Proposition 20.

(1) By Theorem 18, we have *V*_*R*_(*a* → *c*, *b* → *d*) ≥ imin{*V*_*R*_(*a*, *b*), *V*_*R*_(*c*, *d*)} = sup⁡_*y*,*z*∈*L*_ *V*_*R*_(*y*, *z*). Obviously, *V*_*R*_(*a* → *c*, *b* → *d*) ≤ sup⁡_*y*,*z*∈*L*_ *V*_*R*_(*y*, *z*). Then, *V*_*R*_(*a* → *c*, *b* → *d*) = sup⁡_*y*,*z*∈*L*_ *V*_*R*_(*y*, *z*).

Hence, *R* ^*a*→*c*^ = *R* ^*b*→*d*^ by Proposition 20. By definition of the operation → on *L*/*R*, we have *R* ^*a*^ → *R* ^*c*^ = *R* ^*a*→*c*^ = *R* ^*b*→*d*^ = *R* ^*b*^ → *R* ^*d*^.

Hence, the operation → is well defined.

(2) Since

(12) VR(a∨c,b∨d) ≥sup⁡ x∈Limin{VR(a∨c,x),VR(x,b∨d)} ≥imin{VR(a∨c,b∨c),VR(b∨c,b∨d)} ≥imin{VR(a,b),VR(c,d)} =sup⁡y,z∈LVR(y,z),

then *V*_*R*_(*a*∨*c*, *b*∨*d*) ≥ sup⁡_*y*,*z*∈*L*_ *V*_*R*_(*y*, *z*). Obviously, *V*_*R*_(*a*∨*c*, *b*∨*d*) ≤ sup⁡_*y*,*z*∈*L*_ *V*_*R*_(*y*, *z*). Hence, *V*_*R*_(*a*∨*c*, *b*∨*d*) = sup⁡_*y*,*z*∈*L*_ *V*_*R*_(*y*, *z*). That is, *R* ^*a*∨*c*^ = *R* ^*b*∨*d*^.

By Proposition 20 and the definition of the operation ∪ on *L*/*R*, we have *R* ^*a*^ ∪ *R* ^*c*^ = *R* ^*a*∨*c*^ = *R* ^*b*∨*d*^ = *R* ^*b*^ ∪ *R* ^*d*^. That is, ∪ is well defined.

(3) Similar to (2).

(4) Since *R* ^*a*^ = *R* ^*b*^, then *V*_*R*_(*a*, *b*) = sup⁡_*y*,*z*∈*L*_ *V*_*R*_(*y*, *z*). As *V*_*R*_(*a*, *b*) = *V*_*R*_(*a*′, *b*′) = sup⁡_*y*,*z*∈*L*_ *V*_*R*_(*y*, *z*), so *R* ^*a*′^ = *R* ^*b*′^. By the definition of ′, we have (*R* ^*a*^)′ = (*R* ^*b*^)′.

Hence, the ′ is well defined.

**Theorem 23 .**

Let *R* be a vague congruence on a lattice implication algebra *L*. Then, (*L*/*R*, ∪, ∩, ′, →) is a lattice implication algebra.

**Proof**

By Proposition 22, we know that the operations →, ∪, ∩, ′ on *L*/*R* are well defined.

First, we prove that *L*/*R* is a complemented lattice with universal bounds $\bar{O}$, $\bar{I}$.

In fact, for the operation ∪, there exists *R* ^*a*^ ∪ *R* ^*b*^ = *R* ^*a*∨*b*^ = *R* ^*b*∨*a*^ = *R* ^*b*^ ∪ *R* ^*a*^. That means the law of commutativity for ∪ holds. Similarly, the associative and idempotent laws hold. Furthermore, we can get the commutative, associative, and idempotent laws for ∩ and the absorptive law for ∪ and ∩. Hence, (*L*/*R*, ∪, ∩) is a lattice with the partial order ⊆ as follows:

(13) Ra⊆Rb iff  Ra⟶Rb=RIiff  a⟶b=Iiff  a≤b.

If *R* ^*a*^⊆*R* ^*b*^, then *a* ≤ *b*. Hence, *b*′ ≤ *a*′; that is, *R* ^*b*′^⊆*R* ^*a*′^. Thus, (*R* ^*b*^)′⊆(*R* ^*a*^)′. Moreover, because ((*R* ^*a*^)′)′ = (*R* ^*a*′^)′ = *R* ^(*a*′)′^ = *R* ^*a*^, then ′ is an order-reversing involution on *L*/*R*. The universal bounds $\bar{O}=R^{O}$, $\bar{I}=R^{I}$.

Sum up the above, (*L*/*R*, ∪, ∩, ′) is a complemented lattice.

Second, we need to prove (*I*_1_)–(*I*_5_), (*l*_1_), (*l*_2_) hold for *L*/*R*. Here, we only prove (*l*_1_); the others can be proved similarly. In fact, there exists

(14) (Ra∪Rb)⟶Rc=R(a∨b)→c=R(a→c)∧(b→c)=Ra→c∩Rb→c=(Ra⟶Rc)∩(Rb⟶Rc).

This means (*l*_1_) holds.

Hence, (*L*/*R*, ∪, ∩, ′, →) is a lattice implication algebra.

**Proposition 24 .**

Let *R* be a vague congruence relation on a lattice implication algebra *L*. The mapping

(15) $$\begin{array}{c} f:L⟶\frac{L}{R} \end{array}$$

defined by, for *a* ∈ *L*,

(16) $$\begin{array}{c} f\left(a\right)=R^{a} \end{array}$$

is a lattice implication homomorphism.

**Proof**

Obviously, *f* is well defined.

For any *x*, *y* ∈ *L*, *f*(*x* → *y*) = *R* ^*x*→*y*^ = *R* ^*x*^ → *R* ^*y*^ = *f*(*x*) → *f*(*y*). So, *f* is an implication homomorphism. As *f*(*x*′) = *R* ^*x*′^ = (*R* ^*x*^)′ = (*f*(*x*))′, *f* is a lattice implication homomorphism.

**Lemma 25 .**

Let *A* be a vague filter of *L* and let *R*_*A*_ be a vague congruence relation induced by *A*. Then, (*R*_*A*_)^*a*^ = (*R*_*A*_)^*b*^ if and only if *t*_*A*_(*a* → *b*) = *t*_*A*_(*b* → *a*) = *t*_*A*_(*I*) and *f*_*A*_(*a* → *b*) = *f*_*A*_(*b* → *a*) = *f*_*A*_(*I*).

**Proof**

Let (*R*_*A*_)^*a*^ = (*R*_*A*_)^*b*^; we have *V*_(*R*_*A*_)^*a*^_(*a*) = *V*_(*R*_*A*_)^*b*^_(*a*), and so

(17) V(RA)a(a)=imin{VA(a⟶a),VA(a⟶a)}=[tA(I),1−fA(I)]=V(RA)b(a)=imin{VA(b⟶a),VA(a⟶b)}=[min⁡{tA(a⟶b),tA(b⟶a)},  min⁡{1−fA(a⟶b),1−fA(b⟶a)}].

Hence,

(18) min⁡{tA(a⟶b),tA(b⟶a)}=tA(I),min⁡{1−fA(a⟶b),1−fA(b⟶a)}=1−fA(I).

It follows that

(19) tA(a⟶b)=tA(b⟶a)=tA(I),fA(a⟶b)=fA(b⟶a)=fA(I).

Conversely, suppose that *t*_*A*_(*a*⟶*b*) = *t*_*A*_(*b*⟶*a*) = *t*_*A*_(*I*), *f*_*A*_(*a*⟶*b*) = *f*_*A*_(*b*⟶*a*) = *f*_*A*_(*I*).

For any *x* ∈ *L*,

(20) VRAa(x) =imin{VA(a⟶x),VA(x⟶a)} ≥imin{imin{VA(a⟶b),VA(b⟶x)}, imin{VA(x⟶b),VA(b⟶a)}} ≥imin{[min⁡{tA(a⟶b),tA(b⟶x)}, min⁡{1−fA(a⟶b),1−fA(b⟶x)}], [min⁡{tA(x⟶b),tA(b⟶a)}, min⁡{1−fA(x⟶b),1−fA(b⟶a)}]} =imin{[tA(b⟶x),1−fA(b⟶x)],[tA(x⟶b),1−fA(x⟶b)]} =imin{VA(b⟶x),VA(x⟶b)} =VRAb(x).

Similarly, we can show that *V*_*R*_*A*_^*b*^_(*x*) ≥ *V*_*R*_*A*_^*a*^_(*x*). Therefore, *V*_*R*_*A*_^*b*^_(*x*) = *V*_*R*_*A*_^*a*^_(*x*); hence, (*R*_*A*_)^*a*^ = (*R*_*A*_)^*b*^.

**Theorem 26 .**

Let *A* be a vague filter of *L*. Define

(21) $$\begin{array}{c} a\;\equiv_{A}\;b⟺{\left(R_{A}\right)}^{a}={\left(R_{A}\right)}^{b}. \end{array}$$

Then, ≡_*A*_ is a congruence relation on *L*.

**Proof**

The proof can be completed easily from Lemma 25.

**Theorem 27 .**

Let *A* be a vague filter of *L* and let *L*/*R*_*A*_ be the corresponding quotient algebra. Then, the map *f* : *L* → *L*/*R*_*A*_ defined by *f*(*a*) = (*R*_*A*_)^*a*^, for any *a* ∈ *L*, is a lattice implication homomorphism and *D*-*ker*⁡(*f*) = *U*(*t*_*A*_; *t*_*A*_(*I*)∩*L*(*f*_*A*_; *f*_*A*_(*I*)), where *D*-*ker*⁡(*f*) = {*x* ∈ *L*∣*f*(*x*) = (*R*_*A*_)^*I*^}.

**Proof**

The first result is obvious from Proposition 24. We only need to prove the second result, that is,

(22) $$\begin{array}{c} D\text{-ker}\left(f\right)=U\left(t_{A};t_{A}\left(I\right)\right)\cap L\left(f_{A};f_{A}\left(I\right)\right). \end{array}$$

In fact,

(23) $$\begin{array}{c} x\in D\text{-ker}\left(f\right)\\ ⟺{\left(R_{A}\right)}^{x}=f\left(x\right)={\left(R_{A}\right)}^{I}\\ ⟺t_{A}\left(x\to I\right)=t_{A}\left(I\to x\right)=t_{A}\left(I\right),\\ f_{A}\left(x\to I\right)=f_{A}\left(I\to x\right)=f_{A}\left(I\right)\\ ⟺t_{A}\left(x\right)=t_{A}\left(I\right),\;\;f_{A}\left(x\right)=f_{A}\left(I\right)\\ ⟺x\in U\left(t_{A};t_{A}\left(I\right)\right)\cap L\left(f_{A};f_{A}\left(I\right)\right). \end{array}$$

**Corollary 28 .**

Let *A* be a vague filter of *L*; then, *L*/*R*_*A*_≅*L*/≡_*A*_.

## 5. Conclusion

Congruence theory plays a very important role in the research of a logic algebra, through which we can obtain much information such as the internal structure and homomorphism image of the logic algebra and furthermore induce some new algebra with more simple structure. The aim of this paper is to develop the congruence relation on lattice implication algebras. First, the concept of vague similarity relation is introduced based on vague relation, and the concept of vague congruence relations is proposed, and properties and equivalent characterizations of vague congruence relation are investigated. Secondly, the relation between the set of vague filters and the set of vague congruences is studied. Finally, we construct a new lattice implication algebra induced by vague congruences, and the homomorphism theorem is given. The methods in this paper can be used in *MV*-algebras, BL-algebras, MTL-algebras, and so forth. Meanwhile, we hope that it will be of great use to provide theoretical foundation to design intelligent information processing systems.
